# ProtBert-BFD token classification improves intrinsically disordered protein region prediction

**DOI:** 10.1093/bib/bbaf631.062

**Published:** 2025-12-12

**Authors:** Nguyen Quoc Khanh Le

**Affiliations:** In-Service Master Program in Artificial Intelligence in Medicine, College of Medicine, Taipei Medical University, Taipei 110, Taiwan; AIBioMed Research Group, Taipei Medical University, Taipei 110, Taiwan

## Abstract

**Background:**

Intrinsically disordered regions (IDRs) lack stable tertiary structures yet are crucial to transcriptional regulation, signal transduction, and molecular recognition. Experimental annotation of IDRs remains limited—only ~25,000 disordered proteins are cataloged compared with hundreds of millions of known sequences—highlighting the need for scalable computational prediction. We present a systematic evaluation of classical, deep, and transformer-based models for IDR prediction and introduce a fine-tuned ProtBert-BFD token classification framework that achieves high accuracy and generalizability in data-limited settings.

**Methods:**

Using manually curated annotations from the DisProt database, redundant sequences were filtered with CD-HIT (<30% similarity) and restricted to ≤526 residues, producing ~700 high-quality sequences. The dataset was partitioned (70:15:15) into training, validation, and test sets. We compared multiple architectures: (1) logistic regression and multilayer perceptron as classical baselines; (2) bidirectional and Seq2Seq LSTMs capturing sequential dependencies; and (3) ProtBert-BFD models leveraging pretrained protein-language embeddings for residue-level classification.

**Results:**

Classical models showed limited predictive power (AUC 0.56–0.69), while LSTM variants improved recall but overfitted due to data imbalance. ProtBert-BFD fine-tuning substantially enhanced accuracy (75.6%), recall (68.4%), and F1-score (64.0%). The token classification variant achieved precision 0.816, recall 0.823, and F1 0.815—surpassing all internal baselines and approaching the state-of-the-art PROFbval model (recall 0.835). Average inference time was only 1.8 s per 530-residue sequence, enabling proteome-scale deployment.

**Conclusion:**

These findings demonstrate that pretrained transformers effectively capture disorder-related sequence contexts and outperform conventional deep learning in low-data regimes. By integrating transfer learning with efficient inference, the ProtBert-BFD token classification model provides a robust framework for large-scale IDR annotation. Future work will expand datasets, incorporate physicochemical descriptors, and analyze error distributions to refine understanding of protein disorder in health and disease.

**References:**

Hu, G., Katuwawala, A., Wang, K. et al. flDPnn: Accurate intrinsic disorder prediction with putative propensities of disorder functions. *Nat Commun* 12, 4438 (2021).

